# Downregulation of Renal G Protein–Coupled Receptor Kinase Type 4 Expression via Ultrasound‐Targeted Microbubble Destruction Lowers Blood Pressure in Spontaneously Hypertensive Rats

**DOI:** 10.1161/JAHA.116.004028

**Published:** 2016-10-06

**Authors:** Hefei Huang, Xiaolong Li, Shuo Zheng, Yue Chen, Caiyu Chen, Jialiang Wang, Haipeng Tong, Lin Zhou, Jian Yang, Chunyu Zeng

**Affiliations:** ^1^ Department of CardiologyDaping HospitalThe Third Military Medical UniversityChongqingChina; ^2^ Department of RadiologyDaping HospitalThe Third Military Medical UniversityChongqingChina; ^3^ Department of NutritionDaping HospitalThe Third Military Medical UniversityChongqingChina; ^4^ Chongqing Institute of Cardiology & Chongqing Cardiovascular Clinical Research CenterChongqingChina; ^5^ Department of CardiologyThe First Affiliated HospitalShantou Medical CollegeShantouChina

**Keywords:** blood pressure, G protein–coupled receptor kinase type 4, kidney, ultrasound‐targeted microbubble destruction, Hypertension, High Blood Pressure, Nephrology and Kidney

## Abstract

**Background:**

G protein–coupled receptor kinase type 4 (GRK4) plays a vital role in the long‐term control of blood pressure (BP) and sodium excretion by regulating renal G protein–coupled receptor phosphorylation, including dopamine type 1 receptor (D_1_R). Ultrasound‐targeted microbubble destruction (UTMD) is a promising method for gene delivery. Whether this method can deliver GRK4 small interfering RNA (siRNA) and lower BP is not known.

**Methods and Results:**

BP, 24‐hour sodium excretion, and urine volume were measured after UTMD‐targeted GRK4 siRNA delivery to the kidney in spontaneously hypertensive rats. The expression levels of GRK4 and D_1_R were determined by immunoblotting. The phosphorylation of D_1_R was investigated using immunoprecipitation. The present study revealed that UTMD‐mediated renal GRK4 siRNA delivery efficiently reduced GRK4 expression and lowered BP in spontaneously hypertensive rats, accompanied by increased sodium excretion. The increased sodium excretion might be accounted for by the UTMD regulation of D_1_R phosphorylation and function in spontaneously hypertensive rats. Further analysis showed that, although UTMD had no effect on D_1_R expression, it reduced D_1_R phosphorylation in spontaneously hypertensive rats kidneys and consequently increased D_1_R‐mediated natriuresis and diuresis.

**Conclusions:**

Taken together, these study results indicate that UTMD‐targeted GRK4 siRNA delivery to the kidney effectively reduces D_1_R phosphorylation by inhibiting renal GRK4 expression, improving D_1_R‐mediated natriuresis and diuresis, and lowering BP, which may provide a promising novel strategy for gene therapy for hypertension.

## Introduction

The kidney is the major organ involved in the regulation of sodium homoeostasis, and it plays a major role in the long‐term control of blood pressure (BP).^1^ Dopamine, a well‐known neurotransmitter in the central nervous system, also plays an autocrine/paracrine regulatory role with respect to sodium and water transport through the occupation of several receptor subtypes.^2^ Dopamine receptors are classified into D_1_‐ and D_2_‐like subtypes based on their structure and pharmacology. D_1_‐like receptors (D_1_R and D_5_R) couple with the stimulatory G protein Gα_S_ and stimulate adenylyl cyclase (AC) activity, whereas D_2_‐like receptors (D_2_R, D_3_R, and D_4_R) couple with the inhibitory G proteins Gα_i_/Gα_o_ and inhibit AC activity.^3^ In particular, the D_1_‐like receptors are responsible for regulating over 50% of renal sodium excretion when salt intake is increased.^2^, ^3^ In hypertensive states, D_1_R‐mediated natriuresis and diuresis are lost owing to hyperphosphorylation, which leads to the uncoupling of D_1_R from G protein and the consequent loss of function.^3^

G protein–coupled receptor kinases (GRKs) comprise a family of 7 serine/threonine protein kinases that specifically phosphorylate and regulate agonist‐activated G protein–coupled receptors (GPCRs).^4^ Specifically, GRK type 4 (GRK4) appears to play a vital role in the long‐term control of BP and in sodium homoeostasis.^5^ Studies have shown that renal GRK4 modulates dopaminergic‐mediated natriuresis.^6^, ^7^ Increased GRK4 activity contributes to the impairment of renal D_1_R function in hypertension. Depletion of renal cortical GRK4 increases sodium excretion and urine volume and lowers BP in spontaneously hypertensive rats (SHRs).^6^ Therefore, suppression of GRK4 in the kidney is an important issue and might be an alternative method for lowering BP in hypertension.

Ultrasound‐targeted microbubble destruction (UTMD) has received the most attention as an approach for transferring genes or medicines into some organs, including the heart, liver, and kidney.^8^, ^9^, ^10^ We investigated whether this method could deliver GRK4 small interfering RNA (siRNA) and lower BP in SHRs. Our results indicate that UTMD‐targeted GRK4 siRNA delivery to the kidney effectively reduces D_1_R phosphorylation by inhibiting renal GRK4 expression, improving D_1_R‐mediated natriuresis and diuresis, and lowering BP, which may provide a promising novel strategy for gene therapy for hypertension.

## Methods

### Materials

The antibodies (GRK4 and D_1_R) were rabbit anti‐rat antibodies and were purchased from Santa Cruz Biotechnology, Inc. (Dallas, TX) and Millipore (Billerica, MA). The phosphoserine antibody was a rabbit anti‐rat antibody and was purchased from Cell Signaling Technology (Beverly, MA). Fenoldopam, a D_1_‐like receptor agonist, was purchased from Sigma (St. Louis, MO). All other chemicals for the various buffers were of the highest purity available and purchased from Sigma or Gibco (Grand Island, NY).

### Preparation of the Microbubbles Carrying GRK4 siRNA

5‐Carboxyfluorescein (5‐FAM)–labeled GRK4 siRNA was synthesized by RIBOBIO (Gangzhou, China) for fluorescence tracing studies. Microbubbles carrying GRK4 siRNA were prepared using poly‐L‐lysine (PLL) and the electrostatic adsorption method.^11^ The PLL solution (1 mg/mL) was prepared with sterile double‐distilled water. The PLL solution was then mixed with the blank microbubbles at a 1:1 (v/v) ratio and incubated at 4°C for 30 minutes. The mixture was then washed twice with PBS to remove any unbound PLL. GRK4 siRNA was added to this microbubble solution at a 1:1 (v/v) ratio, and the mixture was incubated at 4°C for 30 minutes. Next, 200 mL of PBS was added to the microbubble mixture, and the suspension was washed by centrifugation twice at 1000*g* for 3 minutes to remove any unbound GRK4 siRNA, after which the microbubbles labeled with both GRK4 siRNA and PLL were obtained.

### Animals and UTMD Treatment

The experimental protocols were approved by The Third Military Medical University Animal Care and Use Committee. SHRs (250–260 g) were purchased from the Animal Centre of The Third Military Medical University (Chongqing, China). Male SHRs (SLRC Laboratory Animals, Shanghai, China) ranging in age from 9 to 16 weeks were fed a regular and normal sodium (0.4% NaCl) rat chow. Food, but not water, was withheld for 24 hours before the study.

The experiments were performed in a thermostatically controlled room at an ambient temperature of ≈24°C to 26°C. The rats were fixed in the left lateral position after satisfactory anesthesia by intraperitoneal injection of 2% pentobarbital sodium at a dose of 50 mg/kg. Only the right kidney of each rat was treated with UTMD. The right kidney was localized and irradiated using the 9L4 linear array probe of an ultrasound imaging system for 5 minutes of continuous irradiation (42 frames per s, mechanical index=0.9, frequency=7.00 MHz). The probe was positioned in a transducer holder at an appropriate place to keep the contralateral kidney without treatment by UTMD in the same ultrasound image. Before the injection, a bottle of microbubbles was shaken for 45 seconds in a custom‐built oscillation apparatus to form a microbubble suspension. Microbubbles carrying GRK4 siRNA and blank microbubbles were injected into the lateral tail vein through a 26‐gauge needle connected to a 1‐mL syringe via a 15‐cm‐long catheter (0.45×15 round wall, long bevel, China), controlled by a syringe pump. The injection was completed within 30 seconds and was followed by 0.5 mL saline that was used to wash the tube. The UTMD treatments were performed every 3 days. Each rat in total received 6 treatments.

BP was measured in the conscious rats at 3‐day intervals using the tail‐cuff method (ML125, Power Lab; AD Instruments, Castle Hill, Australia). In brief, before the measurements, the rats were warmed for 15 minutes at 35°C to allow for the detection of tail artery pulsations and to acquire the pulse level. Next, the rats were placed in individual plastic restrainers, and a cuff with a pneumatic pulse sensor was wrapped around their tails. The measurements of arterial BP (systolic, diastolic, and the mean) were performed at least 5 times for each animal, and the mean values of several successive measurements were used. To minimize stress‐induced fluctuations in BP, all of the rats were trained by measuring BP daily for at least 7 days before the beginning of the measurements. The same person acquired the final values in the same peaceful environment. Urine was collected in metabolic cages, and the 24‐hour urine volumes and sodium excretions were also measured at the indicated times. Serum urea nitrogen (BUN) and creatinine levels were estimated using an automated biochemistry machine (Technicon RA‐1000; Bayer, Tarrytown, NY) according to the standard procedure of kits.

### Surgical Procedures and Experimental Protocol for Renal Function Studies

The surgical procedures and surgical interventions were performed as reported.^12^, ^13^ Prior to the performance of the experiments, the rats were anesthetized with pentobarbital (50 mg/kg body weight, intraperitoneally), placed on a heated table to maintain a rectal temperature between 36°C and 37°C, and tracheotomized (PE‐240). Anesthesia was maintained via the infusion of pentobarbital sodium at 0.8 mg/100 g body weight per hour. Catheters (PE‐50) were placed into the external jugular and femoral veins for fluid replacement and the carotid artery for monitoring of the systemic arterial pressure (Cardiomax II; Columbus Instruments, Columbus, OH). A laparotomy was performed, and both the right and left ureters were catheterized (PE‐10). The right renal artery was exposed, and the right suprarenal artery, which originates from the right renal artery, was catheterized (PE‐10 heat stretched to 180 μm) for vehicle (saline) or reagent infusion at the rate of 40 μL/h. The total duration of the surgical procedures was ≈60 minutes. The fluid losses during surgery were replaced with 5% albumin in normal saline at 1% body weight over 30 minutes. After an equilibration period of 120 minutes following the surgery, 6 consecutive 40‐minutes urine samples were collected, including the following: C1 and C2 (averaged as the control period); D1 and D2, (averaged as the fenoldopam period); and R1 and R2 (averaged as the recovery period). During the basal and recovery periods, only saline alone was infused. During the fenoldopam period, fenoldopam (1.0 mg/kg per minute in saline, Tocris Bioscience), which is a D_1_‐like receptor agonist, was infused. The urine was stored at −80°C until use.

### Masson Staining

Samples of kidney were fixed in 4% paraformaldehyde buffer, embedded in paraffin, and cut into 3‐μm sections. The tissue samples were stained using the following steps: stained in Weigert's hematoxylin working solution; added Biebrich scarlet solution; differentiated in phosphotungstic/phosphomolybdic acid solution; added aniline blue solution; differentiated in 1% acetic acid; then the stained tissues were dehydrated, cleared, and mounted on a coverslip. After the Masson staining, the sections were photographed (DN100, E600; Nikon Co., Tokyo, Japan). The fraction of fibrotic tissue was determined using Image J software (NIH, Bethesda, MD).

### Cell Culture

Immortalized renal proximal tubule (RPT) cells from SHRs were cultured at 37°C in a 95% air/5% CO_2_ atmosphere in DMEM/F‐12 culture media as previously described.^14^, ^15^ The cells (80% confluence) were lysed in ice‐cold lysis buffer (PBS with 1% NP40, 0.5% sodium deoxycholate, 0.1% SDS, 1 mmol/L EDTA, 1 mmol/L EGTA, 1 mmol/L PMSF, 10 μg/mL aprotinin, and 10 μg/mL leupeptin), sonicated, maintained on ice for 1 hour, and centrifuged at 16 000*g* for 30 minutes. The supernatants were stored at −70°C until use for immunoblotting.

### Small Interfering RNA

The cells were grown in 6‐well plates until 60% confluence, and 50 nmol/L siRNA or control RNA was mixed with 6 μL of Lipofectamine RNAiMax (Invitrogen, Carlsbad, CA) according to the manufacturer's instruction. Cells were incubated for 25 minutes and then switched to growth medium and incubated for another 24 to 72 hours. Next, the cells were harvested for RNA or protein extraction, which was quantified by real‐time reverse transcription‐quantitative polymerase chain reaction (RT‐qPCR) or Western blotting to determine the efficiency of the siRNA‐induced GRK4 gene silencing. We checked 3 rat GRK4 siRNA sequences including the following: #1 forward primer: 5′‐CCUGUAUUCUUAGACCAAAdTdT‐3′, reverse primer: 3′‐dTdT GGACAUAAGAAUCUGGUUU‐5′; #2 forward primer: 5′‐GGCUGUCUGAUAUAUGAAAdTdT‐3′, reverse primer: 3′‐dTdTCCGACAGACUAUAUACUUU‐5′; #3 forward primer: 5′‐GGAGAGAGCUCCUGAAGUUdTdT‐3′, and reverse primer: 3′‐dTdTCCUCUCUCGAGGACUUCAA‐5′. Next, we selected the most effective primer for the further experiment.

### Immunoprecipitation

Equal amounts of kidney homogenate (800 μg protein) were incubated with affinity‐purified anti‐D_1_R antibody (2 μg) for 1 hour. Next, 50 μL of protein G beads were added, and the mixture was incubated with rocking overnight at 4°C. The immunoprecipitates were pelleted and washed 4 times with lysis buffer. The pellets were suspended in sample buffer, boiled for 10 minutes, and subjected to immunoblotting with polyclonal anti‐phosphoserine antibody.^16^, ^17^

### Quantitative RT‐PCR

For the RT‐qPCR analysis, the cDNA was synthesized from 0.5 mg of total RNA with a cDNA synthesis kit (High Capacity RNA‐to‐cDNA Kit; Takara, Tokyo, Japan). In a thermal cycle, 2 μL cDNA was used per 25‐μL final reaction volume. The PCRs were performed with the Brilliant SYBR Green QPCR Master Mix kit (High Capacity RNA‐to‐cDNA Kit; Takara) in a total volume of 25 μL. For the GRK4, the forward primer was 5′‐TGTCCTGATCCTGAGGC‐3′ and the reverse primer was 5′‐ACACACCCTGTCGCAAT‐3′. For the GAPDH, the forward primer was 5′‐GACATGCCGCCTGGAGAAAC‐3′ and the reverse primer was 5′‐AGCCCAGGATGCCCTTTAGT‐3′. The amplification profile for GRK4 and GAPDH was 95°C for 3 minutes followed by 40 cycles of 95°C for 10 seconds and 60°C for 30 seconds. The RT‐qPCR experiments were repeated 3 times.

### Immunoblotting

After measuring the protein concentration, the supernatants were mixed with Laemmli sample buffer, boiled for 5 minutes, subjected to electrophoresis, and then transferred electrophoretically onto nitrocellulose membranes. The transblots were probed with GRK4 (1:400; Santa Cruz Biotechnology, Dallas, TX) or D_1_R antibody (1:400; Santa Cruz Biotechnology) overnight at 4°C. The membranes were then further incubated with IRDye 800 infrared‐labeled donkey anti‐rabbit secondary antibodies (Li‐Cor Biosciences, Lincoln, NE) at room temperature for 1 hour. The bound complex was detected using the Odyssey Infrared Imaging System (Li‐Cor Biosciences). The images were analyzed using the Odyssey Application Software to obtain the integrated intensities.

### Statistical Analysis

The data are expressed as mean±SEM. First, we addressed the normality assumption for ANOVA and *t* tests and found that the variable was normally distributed. Comparisons within groups were then performed with ANOVA for repeated measures (or paired *t* tests when only 2 groups were compared), and the comparisons among groups (or *t* tests when only 2 groups were compared) were made with ANOVA with Duncan's test. A *P* value *<*0.05 was considered significant.

## Results

### Effective Targeting of GRK4 With RNAi

**Figure 1:**
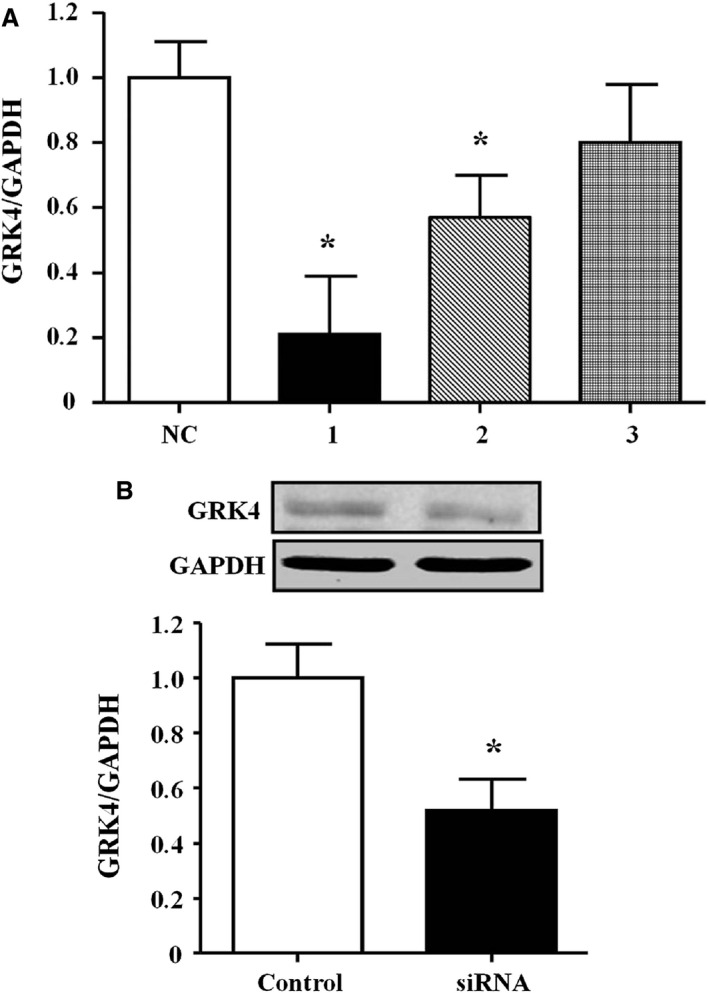
Effects of RNA interference on G protein–coupled receptor kinase type 4 (GRK4) expression in spontaneously hypertensive rat (SHR) renal proximal tubule (RPT) cells. A, GRK4 mRNA expression in SHR RPT cells treated with three GRK4 siRNAs (n=4, *P<0.05 vs control). NC indicates normal control. B, GRK4 protein expression in the #1 GRK4 small interfering RNA (siRNA)–treated SHR PRT cells (n=4, *P<0.05 vs control).

To achieve the expected gene silencing effect of the RNA interference (RNAi), we designed 3 GRK4 siRNAs and then screened for the best. The results revealed that all 3 of the GRK4 siRNAs led to decreased GRK4 mRNA expression in the SHR RPT cells (Figure 1A). However, the #1 GRK4 siRNA had the best effect on GRK4 expression compared with the others (Figure 1B). Therefore, we chose the #1 GRK4 siRNA for the subsequent experiments.

### Physical Properties of Microbubbles Carrying GRK4 siRNA

**Figure 2:**
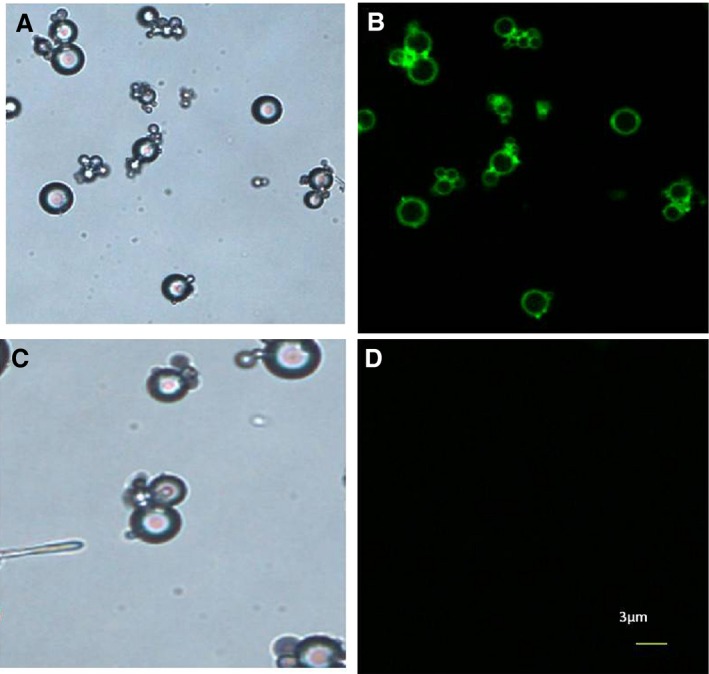
Microbubbles with 5‐Carboxyfluorescein (5‐FAM)–labeled G protein–coupled receptor kinase type 4 (GRK4) small interfering RNA (siRNA) observed under fluorescence microscopy. Microbubbles conjugated with 5‐FAM–labeled GRK4 siRNA were observed under bright‐field microscopy (A) and fluorescence microscopy (B). Only the microbubbles conjugated with 5‐FAM–labeled GRK4 siRNA exhibited green fluorescence. Blank microbubbles under bright‐field microscopy (C). No fluorescence was observed in the blank microbubbles under fluorescence microscopy (×400) (D).

Images obtained under bright‐field microscopy revealed that the microbubbles with 5‐FAM‐labeled GRK4 siRNA were homogeneous in size and distributed evenly with no significant aggregation (Figure 2A). Fluorescence microscopy confirmed that the siRNA labeling approach was effective, and only the microbubbles conjugated with the 5‐FAM‐labeled GRK4 siRNA exhibited green fluorescence (Figure 2B). No fluorescence was observed from the control microbubbles (Figure 2C and 2D).

### UTMD‐Mediated Delivery of GRK4 siRNA Decreases BP in SHRs

**Figure 3:**
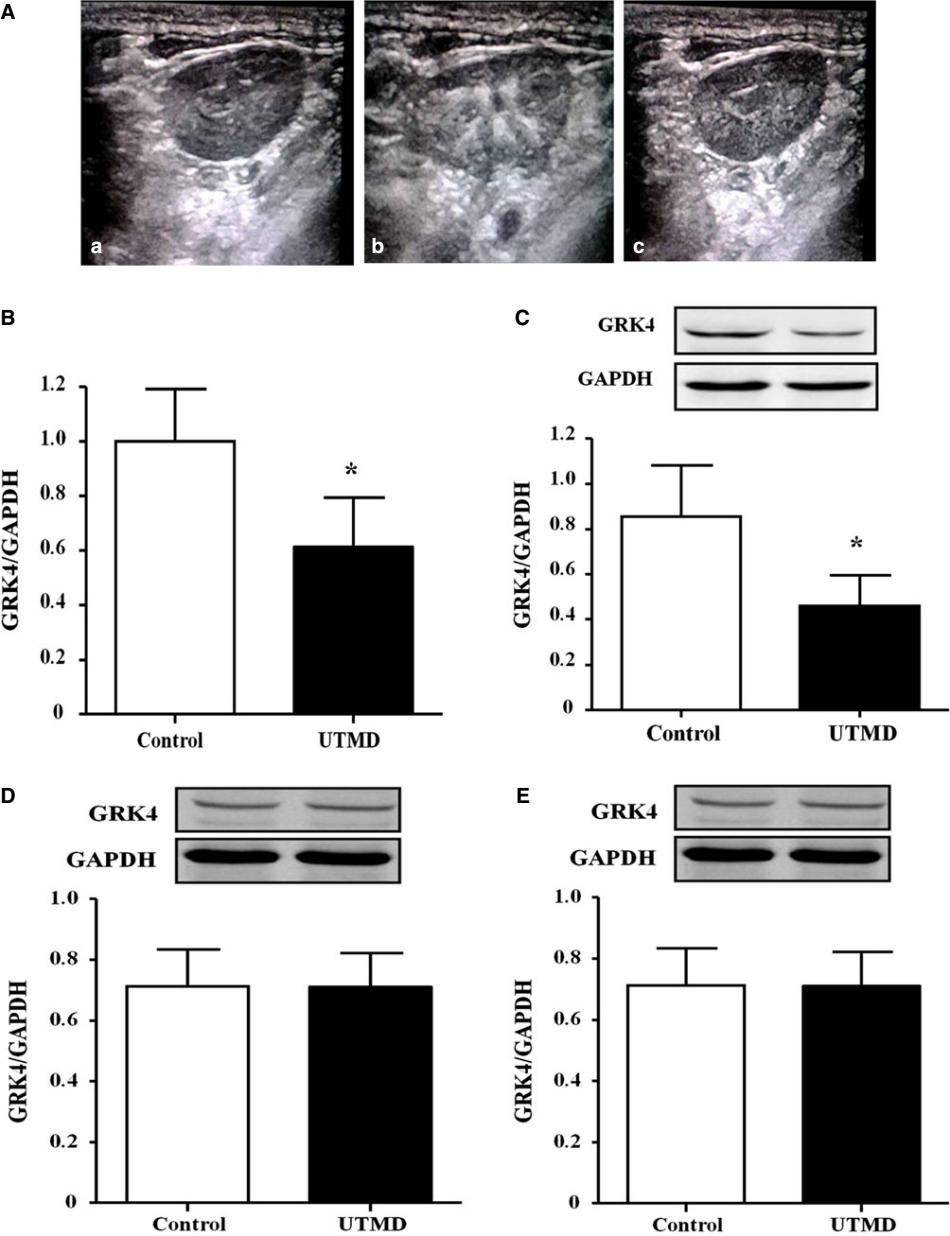
Inhibitory effects of ultrasound‐targeted microbubble destruction (UTMD)–mediated G protein–coupled receptor kinase type 4 (GRK4) small interfering RNA (siRNA) delivery in renal GRK4 expression. A, Ultrasound image of the kidney of a spontaneously hypertensive rat (SHR) (42 frames per s, mechanical index=0.9, frequency=7.00 MHz). B‐mode scans, gray‐scale mapping. The images were obtained at 0 seconds (a), 15 seconds (b), and 5 minutes (c) after the injection of the microbubbles. B and C, Renal GRK4 mRNA and protein expression levels after treatment with UTMD‐mediated GRK4 siRNA delivery for 20 days in SHRs (n=5, *P<0.05 vs control). D and E, Effects of UTMD‐mediated GRK4 siRNA delivery on GRK4 expression in the heart and mesenteric arteries of SHRs after treatment with UTMD‐mediated GRK4 siRNA delivery for 20 days (n=5).

Figure 3A‐a demonstrates a renal ultrasound image in one SHR. Renal opacification and subsequent microbubble destruction were observed under ultrasonic irradiation, which indicated that the renal perfusion was sufficient (Figure 3A‐b and 3A‐c). After treatment with UTMD for 20 days, both the renal GRK4 mRNA and protein expression levels were found to be markedly decreased (Figure 3B and 3C). To exhibit the specificity of the UTMD, we also checked the GRK4 expression in the heart and arteries, which resulted in UTMD‐mediated GRK4 siRNA delivery that had no effect on GRK4 expression in those tissues (Figure 3D and 3E).

**Figure 4:**
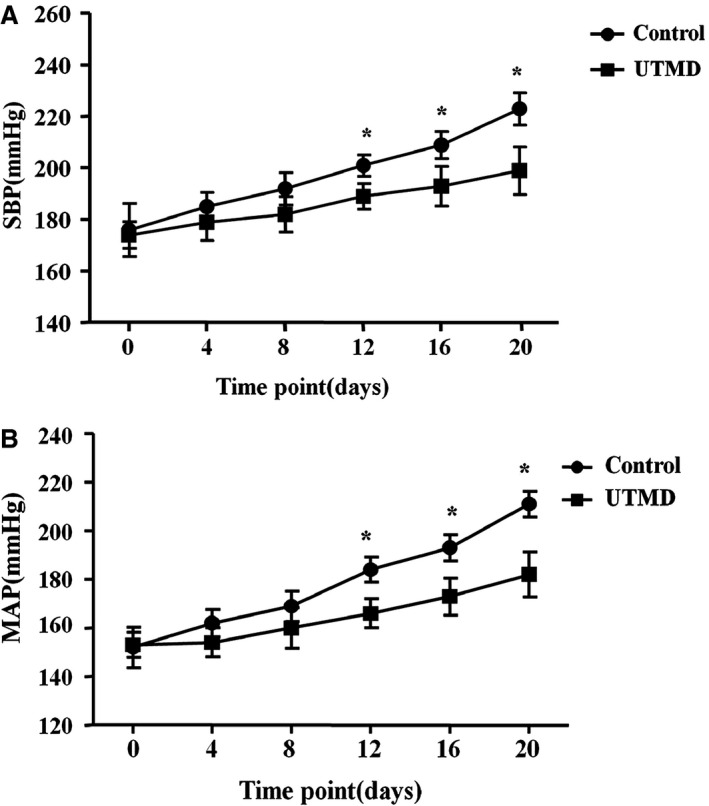
Effects of ultrasound‐targeted microbubble destruction (UTMD)–mediated G protein–coupled receptor kinase type 4 (GRK4) small interfering RNA delivery on blood pressure. UTMD‐mediated GRK4 small interfering RNA delivery decreased systolic blood pressure (SBP) (A) and mean arterial pressure (MAP) (B) in spontaneously hypertensive rats (n=5, *P<0.05 vs control).

**Figure 5:**
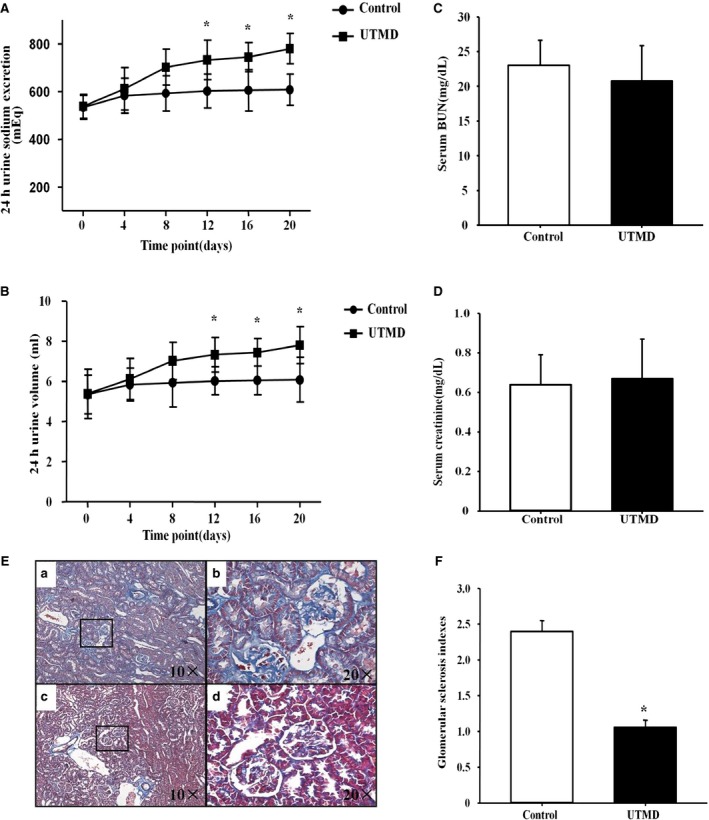
Effects of ultrasound‐targeted microbubble destruction (UTMD)–mediated G protein–coupled receptor kinase type 4 (GRK4) small interfering RNA (siRNA) delivery on sodium excretion. A and B, Sodium excretion (A) and urine volume (B) of spontaneously hypertensive rats (SHRs) were recorded after UTMD treatment (n=5, *P<0.05 vs control). C and D, Renal function (C, serum urea nitrogen [BUN]; D, serum creatinine) of the SHRs after treatment with UTMD‐mediated GRK4 siRNA for 20 days. E, Representative photographs of Masson trichrome staining of the UTMD‐mediated GRK4 siRNA deliveries in the kidneys of SHRs compared with controls. a, Glomerulosclerosis was evident in the SHR controls. b, The ×20 amplification of the black frame of figure (a). c, Significantly decreased fibrotic areas in the UTMD‐mediated GRK4 siRNA deliveries in the SHRs. d, The amplified part in the black frame of figure (c). F, The renal glomerular sclerosis indices for Masson staining (n=5, *P<0.05 vs control).

Owing to the importance of GRK4 on BP regulation, we also checked BP in those rats. The results revealed that the basal BP was comparable between the UTMD treatment group and the control group. A significant difference was found from 12 days after UTMD treatment and was maintained for at least 20 days (Figure 4A and 4B). Our further study also revealed that GRK4 siRNA via UTMD increased the 24‐hour sodium excretion and urine volume from 12 days (Figure 5A and 5B).

We also measured renal function and the effect on renal fibrosis after UTMD‐targeted GRK4 siRNA delivery to the kidneys in the SHRs. The results revealed that there were no differences in BUN or serum creatinine levels between the UTMD treatment group and the control group (Figure 5C and 5D). However, renal fibrosis was decreased after treatment with UTMD for 20 days in the SHRs (Figure 5E and 5F).

### UTMD‐Mediated Delivery of GRK4 siRNA Regulates D_1_R Phosphorylation and D_1_R‐Mediated Natriuresis in SHRs

**Figure 6:**
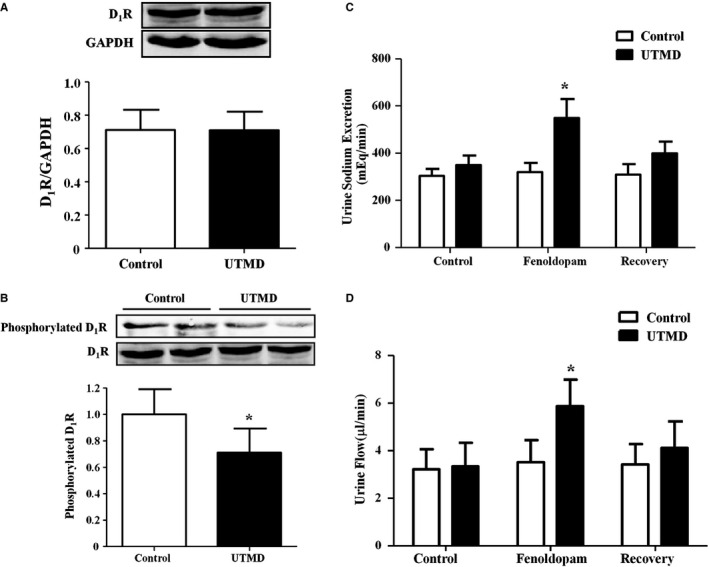
Effects of ultrasound‐targeted microbubble destruction (UTMD)–mediated G protein–coupled receptor kinase type 4 (GRK4) small interfering RNA (siRNA) delivery on dopamine type 1 receptor (D_1_R) phosphorylation and D_1_R‐mediated natriuresis. A and B, Effects of UTMD‐mediated GRK4 siRNA delivery for 20 days on renal D_1_R expression (A) and phosphorylation (B) in spontaneously hypertensive rats (SHRs) (n=5, *P<0.05 vs control). C and D, Effects of fenoldopam, a D_1_‐like receptor agonist, on sodium excretion and urine volumes in the UTMD (20 days)‐treated SHRs (n=5, *P<0.05 vs control).

As mentioned above, D_1_R is the major target of GRK4 in the kidney. We determined whether GRK4 siRNA via UTMD in the kidney regulates D_1_R phosphorylation and function in SHRs. Our results revealed that, although there was no difference in the D_1_R protein expression between the UTMD treatment group and the control group (Figure 6A), GRK4 siRNA via UTMD decreased the phosphorylation of D_1_R compared with the control group (Figure 6B). Moreover, UTMD‐mediated delivery of GRK4 siRNA also led to an improvement in D_1_R‐mediated natriuresis in SHRs because fenoldopam, a D_1_‐like receptor agonist, led to a greater increase in sodium excretion and urine volume in the UTMD treatment group than the control group (Figure 6C and 6D). These results suggest that silencing GRK4 expression via UTMD improves renal D_1_R function.

## Discussion

It is well acknowledged that dopamine plays a vital role in the regulation of BP by functioning as an autocrine/paracrine regulator of sodium and water transport via the occupation of 5 receptor subtypes. All 5 dopamine receptors are present in the kidney. The disruption of any of the dopamine receptor genes in mice results in hypertension, which further indicates the role of the dopamine receptor in the regulation of BP.^18^, ^19^ During normal or moderately increased NaCl intake, inhibition of D_1_‐like receptors decreases sodium excretion by ≈60%^20^, ^21^ Activation of D_1_‐like receptors can inhibit sodium hydrogen exchanger type 3, sodium phosphate cotransporter type IIa, the chloride bicarbonate exchanger, probably the NaCl cotransporter, and the epithelial sodium channel at the luminal membrane in addition to Na^+^‐K^+^ ATPase and the sodium bicarbonate cotransporter at the basolateral membrane.^22^, ^23^, ^24^ The ability of dopaminergic reagents to inhibit renal ion transport is impaired in humans with essential hypertension and rodents with genetic hypertension.^25^, ^26^ The impaired renal tubular dopaminergic function in hypertension has been ascribed to the hyperphosphorylation of dopamine receptors, especially D_1_R, and their uncoupling from their G protein/effector complex, which consequently leads to an impaired inhibitory effect on renal ion transport, decreased dopamine‐mediated natriuresis and diuresis, and increased BP.^27^, ^28^

GRKs, including GRK1‐GRK7, interact with the agonist‐activated GPCRs to promote receptor phosphorylation and initiate receptor desensitization.^4^ In the process of receptor desensitization, GRKs phosphorylate agonist‐bound GPCRs, which causes the translocation and binding of arrestins to GPCRs and the inhibition of the subsequent receptor activation via the blocking of GPCR‐G protein coupling. Increasing numbers of studies have shown that GRKs are associated with hypertension and BP responses to antihypertensive medicines.^29^, ^30^ In particular, GRK4 seems to play a vital role in regulating dopamine‐mediated natriuresis.^31^, ^32^ GRK4 activity is increased in the kidneys of humans with essential hypertension.^7^ Basal GRK4 expression and serine‐phosphorylated D_1_R levels are much higher in SHRs than in Wistar‐Kyoto (WKY) rats.^6^ Silencing of renal cortical GRK4 decreases serine‐phosphorylated D_1_R in both SHRs and WKY rats.^6^ In human RPT cells, GRK4 constitutively phosphorylates D_1_R in the absence of agonist activation; however, both heparin, an inhibitor of GRK activity, and GRK4 antisense oligonucleotides blunt the desensitization of D_1_R,^33^ which recovers the impaired D_1_R‐mediated natriuresis and diuresis. In addition to the regulation of D_1_R, GRK4 has also been found to regulate the phosphorylation and function of renal D_3_R.^34^ As previously mentioned, the inhibition of GRK4 expression restores dopamine receptor function and lowers BP. Therefore, the selection of better approaches to suppress renal GRK4 expression is an important issue for the regulation of sodium excretion and BP in hypertension. However, currently, it is difficult to specifically inhibit GRK4 activity because there is no specific inhibitor for GRK4. The limitations to the current methods for inhibiting GRK4 are obvious.^33^, ^34^, ^35^, ^36^ For example, heparin is not a specific inhibitor of GRK4. The gene transfection efficiency is lower because of the ubiquitous RNAse.^33^, ^34^, ^35^, ^36^ Moreover, the use of a virus for gene transfection would lead to safety concerns. Therefore, a nonviral and highly efficient method would be desirable in clinics.

Microbubbles, which are an emerging nonviral vector system for the delivery of siRNA, may overcome the current siRNA delivery limitations.^35^ Currently, UTMD‐mediated gene and drug delivery have been widely used in studies on cardiovascular disease.^36^, ^37^, ^38^ However, there are no reports of evaluations of UTMD‐mediated gene delivery related to the treatment of hypertension. In the present study, we found that UTMD‐targeted GRK4 siRNA delivery reduced renal GRK4 expression and consequently decreased BP in SHRs, which suggests that UTMD‐mediated siRNA delivery is an efficient method for the delivery of siRNAs to the kidney and a potential clinical strategy for lowering BP in the future.

In addition, we also observed the protection of UTMD‐targeted GRK4 siRNA delivery to the renal function. Our present study found that, although the renal fibrosis is significantly different between SHRs and the UTMD‐targeted GRK4 siRNA–treated SHRs, the renal function, determined by BUN and creatinine, has no difference between two groups. The reasons leading to the different results for fibrosis and renal function are not completely known; however, at least, the compensatory effect of residual normal nephron may be involved. In fact, a similar phenomenon has been reported in a previous study. SHRs do not show a difference when compared with WKY rats regarding serum BUN or creatinine levels.^39^ However, renal fibrosis is increased in SHRs compared with WKY rats.^40^ Although the mechanisms leading to renal fibrosis are complicated, local factors, such as angiotensin II and transforming growth factor‐β, have been demonstrated to be involved in the pathogenesis of renal fibrosis. Our previous studies have shown that inhibition of GRK4 reduced renal and arterial angiotensin II type 1 receptor (AT_1_R) expression.^17^, ^41^ Therefore, UTMD‐targeted GRK4 siRNA delivery may also decrease AT_1_R‐mediated profibrogenic effects via reducing AT_1_R expression, which needs to be determined in the future.

## Conclusions

**Figure 7:**
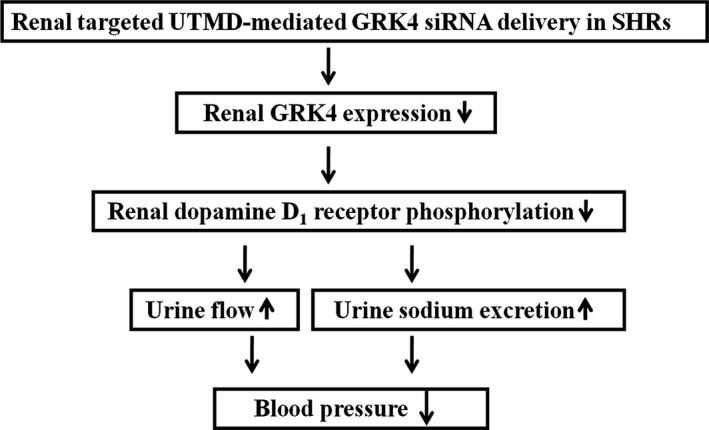
Schematic representation of the effects of ultrasound‐targeted microbubble destruction (UTMD)–mediated G protein–coupled receptor kinase type 4 (GRK4) small interfering RNA (siRNA) delivery on blood pressure in spontaneously hypertensive rats (SHRs).

The present study demonstrates that in SHRs, UTMD‐targeted GRK4 siRNA delivery to the kidney reduces GRK4 expression, restores D_1_R phosphorylation and associated sodium excretion, and leads to lowered BP (Figure 7). These findings indicate that UTMD‐mediated GRK4 siRNA delivery may provide a promising novel strategy for gene therapy for hypertension.

## Sources of Funding

These studies were supported in part by grants from the National International Technology Special Grant (2014DFA31070), the National Natural Science Foundation of China (31430043, 81500536), and the Natural Science Foundation Project of Chongqing (cstc2015jcyjA10060).

## Disclosures

None.
